# Observation of Microstructure Formation During Freeze-Drying of Dextrin Solution by *in-situ* X-ray Computed Tomography

**DOI:** 10.3389/fchem.2018.00418

**Published:** 2018-09-14

**Authors:** Kyuya Nakagawa, Shinri Tamiya, Shu Sakamoto, Gabsoo Do, Shinji Kono

**Affiliations:** ^1^Division of Food Science and Biotechnology, Graduate School of Agriculture, Kyoto University, Kyoto, Japan; ^2^College of Bioresource Science, Nihon University, Fujisawa, Japan; ^3^Research and Development Center, Mayekawa MFG. Co., Ltd., Tokyo, Japan

**Keywords:** X-ray computed tomography, ice microstructure, freeze-drying, annealing, dextrin

## Abstract

*In-situ* X-ray computed tomography (CT) was used to observe microstructure formations during freeze-drying of a dextrin solution. A specially designed freeze-drying stage was equipped at the X-ray CT stage. Frozen and dried microstructures were successfully observed. The CT images of the frozen solution clarified the ice crystal size increase and obvious boundary formation between the ice and freeze-concentrated phases upon performing post-freezing annealing at −5°C. These structural modifications emerged owing to Ostwald ripening and glassy phase relaxation. During the freeze-drying, pore microstructures formed as a consequence of water removal. The pores were replicas of the original ice microstructures; some pore microstructures newly formed by the removal of water. The latter mechanism was more obvious in the non-annealed sample than in the annealed sample. The glassy phase in the non-annealed solution was not perfectly freeze-concentrated; water was rapidly removed from this phase, losing its original microstructure. At this moment, the freeze-concentrated region piled up to new pore walls, which consequently thickened the pore walls. An image analysis estimated that the mean pore wall thicknesses for the non-annealed and annealed samples were 13.5 and 8.6 μm, respectively. It was suggested that the advantages of annealing are not only to reduce drying time owing to the modification of ice crystal morphologies but also to avoid quality loss related to the structural deformation of the glassy matters.

## Introduction

Freeze-drying is known as one of the best drying methods in terms of preservation of product qualities. Owing to its high operation and investment costs, quality assurance is a critical issue to meet industry requirements. Detailed knowledge of phenomena occurring during freeze-drying is useful for further improvement of the process design. The freeze-drying process can be mainly separated into freezing and drying steps. When an aqueous solution is subjected to freezing, an ice crystal phase coincidently forms with the freeze-concentrated phase. In most cases of pharmaceutical and food products, formulations contain compounds such as sugar, protein, polysaccharide, peptide, oligomer, etc. When aqueous solutions of these compounds (or mixtures) are freeze-concentrated, this phase consequently transforms into a glassy state at a maximally concentrated level. Water acts as a plasticizer in a glassy system; the viscosity (or plasticity) of the glassy state is a function of the temperature and water content. The viscosity of the glassy state increases over 10^12^ Pa·s below the glass transition temperature (*T*_g_). The glass transition temperature of the maximally freeze-concentrated glassy phase is commonly denoted as *T'*_g_. This temperature is crucial in the operation of a freeze-drying run. *T'*_g_ is the highest temperature achieving perfect solidification (i.e., non-equilibrium solidification) of a formulation. When a formulation is rapidly frozen, the glassy phase is not perfectly freeze-concentrated owing to the formation of non-crystalized water (i.e., vitrified water) (Levine and Slade, 1988; Ablett et al., 1992; Sahagian and Goff, 1994; Kawai et al., 2006; Ohkuma et al., 2008). Annealing over *T*_g_ reduces the fraction of amorphous phase, thus achieving completion of the freeze-concentration and leading to a reduction in the water content. This phenomenon is known as glassy state relaxation, where the state behavior is governed by the mobility of the matrix plasticized by water. On the other hand, ice crystals coexisting with the glassy phase can recrystallize above *T'*_g_ to minimize the interphase surface area, leading to ripening of ice crystals. This phenomenon is referred to as Ostwald ripening (Kurz and Fisher, 1986; Ratke and Voorhees, 2013), which modifies ice microstructures in terms of size and uniformity (Searles et al., 2001).

During the drying step, water is mainly removed from the ice crystal phase by sublimation (i.e., primary drying). The removal of water coincidently forms a dried layer; the subsequently sublimated water vapor must transfer through the dried cake layer with porous microstructures. Therefore, the mass transfer resistance in the cake layer is controlled by modifying the ice crystal morphologies (Searles et al., 2001; Nakagawa et al., 2006, 2007). Water is also removed from the glassy phase by vaporization (i.e., secondary drying) above *T'*_g_. The viscous flow of the glassy phase may cause loss of the dried layer structure, referred to as collapse. Therefore, the collapse is provoked by the excessive increase of the product temperature determined by the water removal rate by both sublimation and evaporation, affected by factors such as ice crystal microstructure, degree of glassy state relaxation, dried-cake-layer strength, product geometry, thermal property, heat transfer mechanism, etc. The complex combination of phenomena during freezing leads to product variances in terms of drying kinetics and resultant properties (Pikal and Shah, 1990; Kasper and Friess, 2011; Gianfrancesco et al., 2012; Andrieu and Vessot, 2017). The degree of glassy state relaxation is related to the *T*_g_ and viscosity of the glassy state; it would affect the dehydration rate from the glassy phase and consequently the strength of the dried layer. Therefore, the drying behavior in the sufficiently freeze-concentrated frozen solution would be different from that in the immature frozen solution. In this study, we investigate the influence of the glassy state relaxation on the microstructure formation during freeze-drying.

X-ray computed tomography (CT) is a powerful method to visualize the microstructure of a material without sample destruction, and quantify structural information based on the obtained images (Schladitz, 2011; N'Diaye et al., 2013; Ndiaye et al., 2015). A CT image is composed of volume elements; the gray levels in the image correspond to the attenuation of X-rays passing through each volume element. The X-ray attenuation is mainly determined by the applied X-ray energy, material density, and composition. In a previous study, X-ray CT was used to observe frozen solutions using monochromatized X-rays from synchrotron radiation (Nakagawa et al., 2018). This study attempted to track the progress of glassy state relaxation based on CT images, and clarified the kinetics of the ice crystal growth by the glassy phase relaxation and Ostwald ripening.

In this study, X-ray CT was used to observe microstructure formation during freeze-drying of dextrin solution. Dextrin is a polysaccharide, mixtures of polymers of D-glucose units, that is a common food additive as a formulation aid, processing aid, stabilizing agent, etc. Dextrin with a desired glass transition properties can be obtained by changing the average degree of polymerization. We focused on the drying behavior using an *in-situ* CT technique with monochromatized X-rays from synchrotron radiation. A specially designed freeze-drying stage was installed at the X-ray CT stage. *In-situ* microstructure observations during vacuum freeze-drying of a dextrin solution were performed.

## Materials and methods

### Materials

Millipore-purified water was used for the sample preparation. Dextrin was donated by Matsutani Chemical Industry Co., Ltd. (Hyogo, Japan), whose dextrose equivalent (DE) was 11. The glass transition temperature of the maximally freeze-concentrated aqueous solution of dextrin (*T'*_g_), measured by DSC, was approximately −11°C.

### Frozen-sample preparation

**Figure 1:**
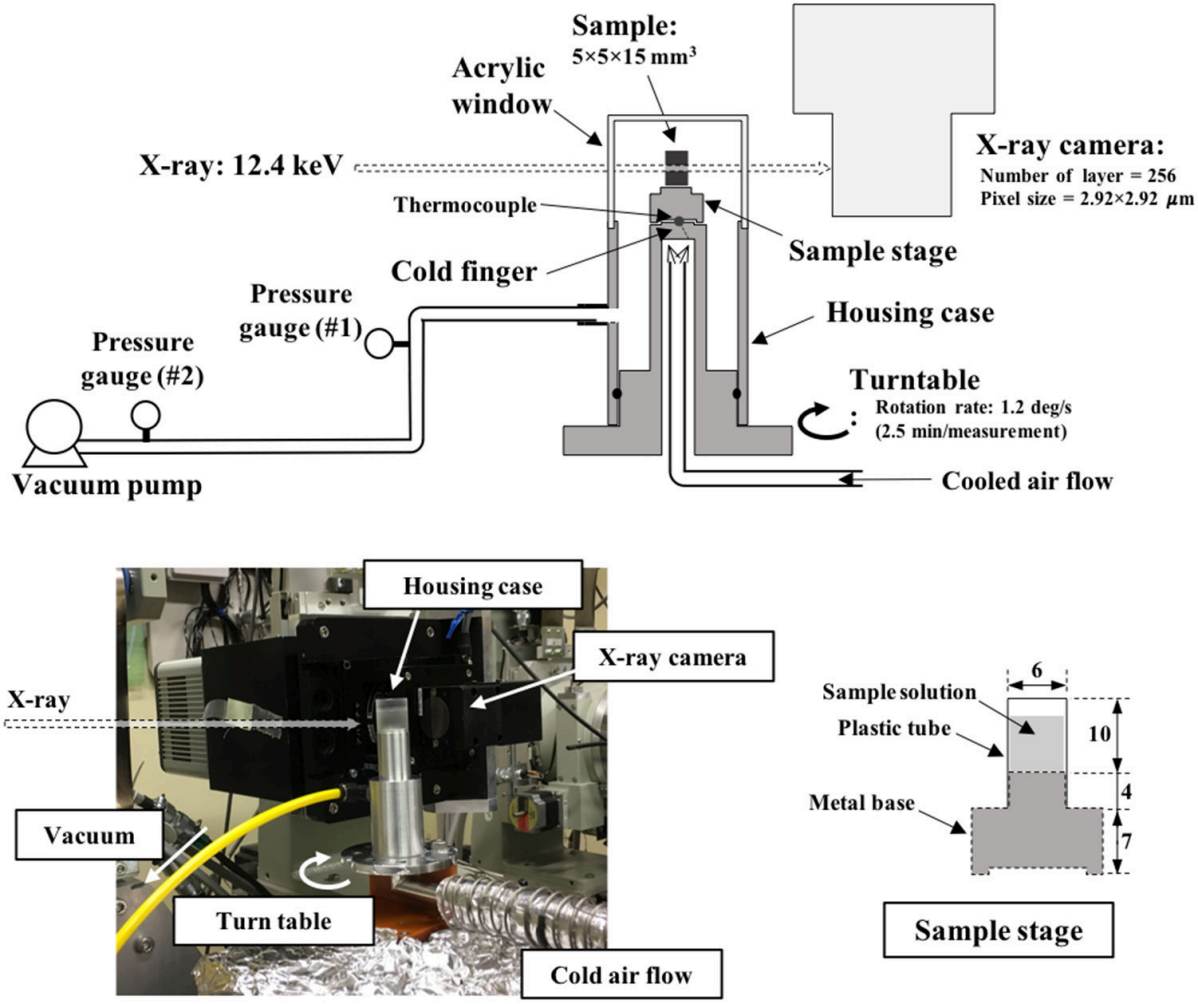
X-ray CT measurement setup equipped with a freeze-drying stage.

An aqueous dextrin solution with a concentration of 20%(w/w) was prepared and filled in a sample stage. The sample stage, illustrated in Figure 1, was composed of a metal base (made of stainless steel) and plastic tube (diameter: 6 mm, height: 10 mm). The tube was attached on the metal base to provide space to insert the solution. The bottom of the solution contacts the metal part. The sample stage with the sample solution was placed on a copper block, pre-cooled in liquid nitrogen, to rapidly freeze the inner solution maintaining its direction of freezing from the bottom to the top. Frozen solutions were subsequently annealed at −5°C in an electric cooling device for a selected duration (0–12 h). The frozen samples were stored in a refrigerator at −90°C until measurement.

### Synchrotron X-ray computed microtomography

X-ray CT was performed at the synchrotron facility SPring-8 (BL19B2, Hyogo, Japan). Monochromatized X-rays were used for this measurement in order to obtain the X-ray attenuation linear coefficient from the tomograms. The X-ray energy was adjusted to 12.4 keV by a double Si-crystal monochromator [net plane: (111)]. As schematized in Figure 1, the measurement stage was equipped with a turn-table with a cold finger whose temperature was controllable by an external air-blowing device in the range of −80°C to −10°C. The sample stage with a frozen sample was rapidly fixed on the cold finger, pre-cooled at approximately −15°C. The sample stage on the turn-table was then fully covered with a housing case, and the inner space was evacuated by connecting a vacuum pump (NeoDry15E, Kashiyama Industries, Ltd., Japan) in order to start the freeze-drying. The maximum evacuation rate of this pump was 0.25 m^3^/min. The top of this housing component was made of acrylic resin with a thickness of 0.5 mm. X-rays passed through this acrylic window and sample, and then exposed the X-ray camera, set 100 mm behind the housing component. An X-ray imaging unit (AA40, Hamamatsu Photonics K.K., Japan) and charge-coupled device (CCD) camera (C4880-41S, Hamamatsu Photonics K.K., Japan) were used for image acquisition. A total of 256 transmission images were acquired in one set of measurement by rotating the turn-table from 0° to 180° at a speed of 1.2°/s. The exposure time of X-rays in the image acquisition was set to 0.12 s; the image pixel size was 2.92 μm (square). A horizontal cross-sectional image (i.e., tomogram) was reconstructed from the transmission image by the filtered back-projection method.

## Results and discussion

### Performance of the freeze-drying system

The freeze-drying performance of the employed system was evaluated by sublimating pure ice before loading on the X-ray CT stage. Distilled water was filled in the sample stage and frozen on the copper block, pre-cooled in liquid nitrogen. The frozen sample was placed on the cold finger pre-cooled at approximately −30°C. The sample stage was then covered with the housing case, and the system was evacuated by connecting the vacuum pump to start the sublimation. The sample stage temperature (*T*_sh_) was adjusted in the range of −20°C to −7°C, while the pressure was monitored with pressure gauges #1 (P1) and #2 (P2). Considering the system limitation, the vacuum line was connected by a flexible tube with a length of approximately 3 m (outer diameter: 6 mm; inner diameter: 4 mm; distance from the housing case to the pressure gauge #1: 1.5 m; distance between the pressure gauges #1 and #2: 1.2 m). A cold trap was not included in the employed setup; instead, a vacuum pump with an evacuation rate of 0.25 m^3^/min was employed. The ratio of the upstream pressure to the downstream pressure could be an indicator of the choked flow of the sublimated water vapor in the system. It was reported that the ratio was larger than 2.5 in the choked-flow regime when a freeze-drying run was operated with a sample load of 5 kg in a laboratory-scale shelf-type freeze-dryer (LyoStar II) (Patel et al., 2010). According to the values presented in Table 1, the employed drying system was operated with a P2/P1 ratio in the range of 2.2–2.8. Assuming that P2/P1 = 2.5 is the threshold value of the choked-flow regime, an operation at approximately *T*_sh_ = −14°C could avoid choked flow. It is worth noting that the employed drying system was operated at conditions quite close to the choked-flow regime.

**Table 1:** Sample stage temperature and inner pressures.

***T*_sh_ [°C]**	**P1 [Pa]**	**P2 [Pa]**
−19.0	7.24	2.64
−14.4	11.8	5.14
−10.8	12.57	4.57
−7.8	14.16	5.14

**Figure 2:**
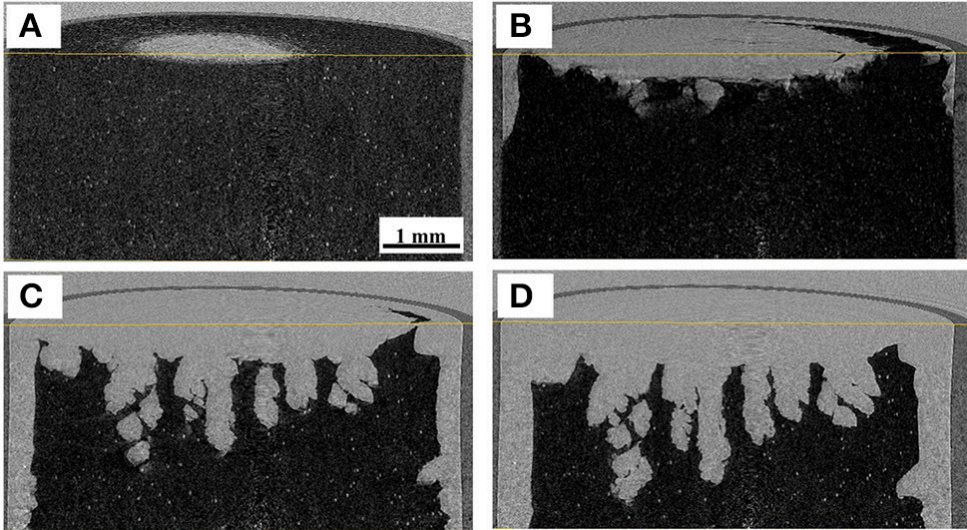
X-ray CT images during ice sublimation, for samples dried for **(A)** 0, **(B)** 600, **(C)** 1,200, and **(D)** 1,800 s.

CT images during ice sublimation are shown in Figure 2. It can be confirmed that the ice sublimation was successfully realized in the employed system. Although the sublimation progressed form the top to the bottom, sublimation from the outer surface was also observed. This suggests that the radiative heat from the housing material was not negligible in this system. The inhomogeneity of the sublimation interface could be explained by the zone sublimation model (Toei et al., 1975). The heat flux delivered from the sample stage to the bottom of the hole was larger than that at the top surface, thus enhancing the interfacial inhomogeneity. The sublimation rate could be calculated from the difference in the ice interface positions between the fixed time interval. The sublimation rate was roughly estimated to be 8.5 × 10^−5^ kg/h, assuming that a 1.5-mm-thick ice layer with a diameter of 6 mm was removed in 1,800 s.

### CT images of frozen samples

**Figure 3:**
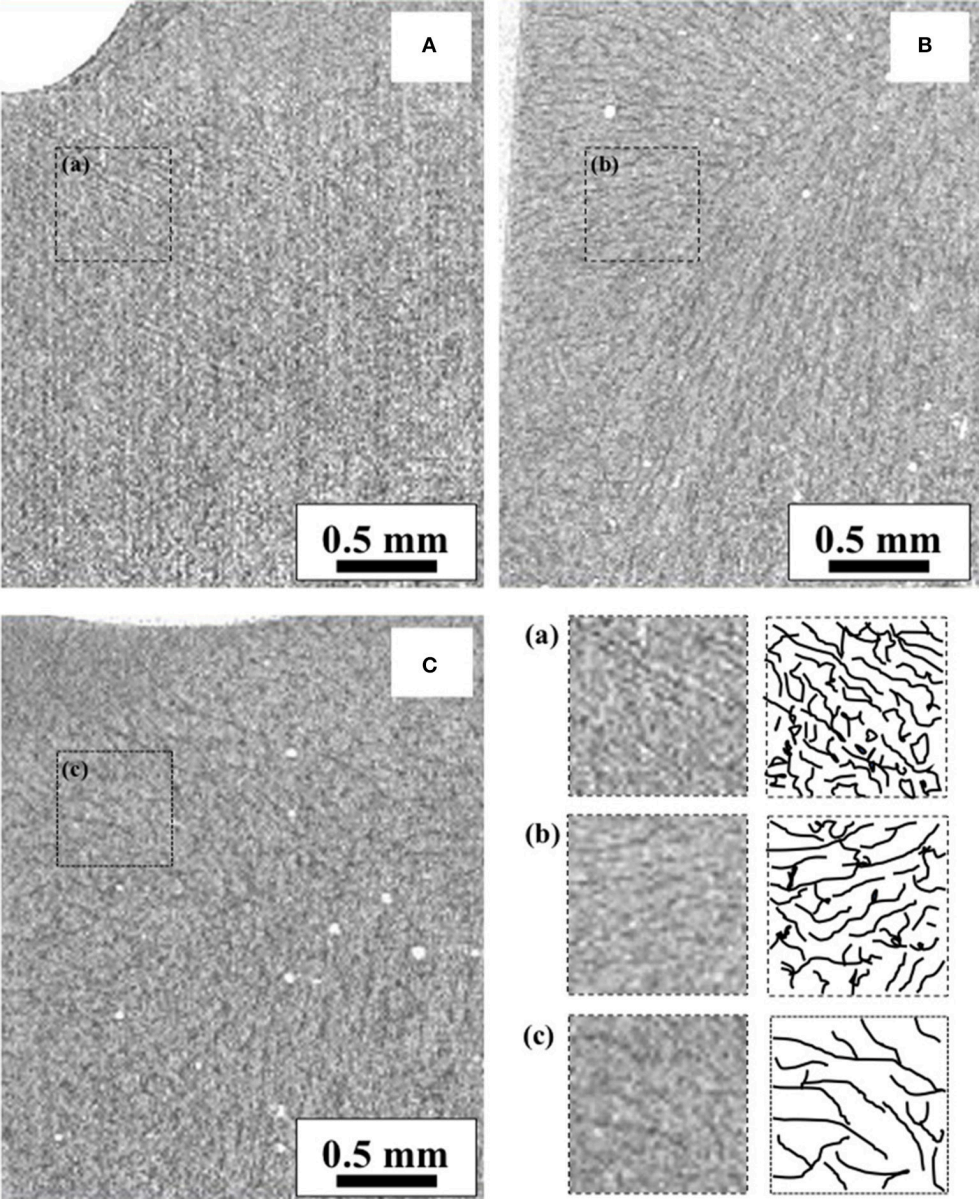
X-ray CT images of frozen samples: **(A)** non-annealed, **(B)** annealed for 12 h, and **(C)** annealed for 35 h.

CT images acquired from the frozen samples (i.e., yet dried samples at the beginning of the freeze-drying run) are compared in Figure 3. In these images, the gray level corresponds to the matrix density; therefore, the dark and shallow areas correspond to the freeze-concentrated and ice crystal phases, respectively. The increase in the ice crystal sizes with the annealing time can be confirmed. The boundary between the ice and freeze-concentrated phases became obvious. The ice crystal size increase could be attributed to Ostwald ripening. Another origin of the increase could be attributed to the progress of the freeze-concentration owing to the glassy state relaxation. The details of these kinetics during annealing were investigated in a previous study (Nakagawa et al., 2018), suggesting that both glassy phase relaxation and Ostwald ripening control the ice crystal growth/ripening kinetics; the dominant mechanism depended on the stage of annealing. The progress of the freeze-concentration increases the density of the phase, which contributes to an enhanced attenuation level in the CT image and provides clear contrast with respect to the ice phase. It was also suggested that the dextrin–water system (with dextrin DE of 11) required more than 20 h of annealing for the completion of the glassy phase relaxation even at −5°C.

### CT images during freeze-drying

**Figure 4:**
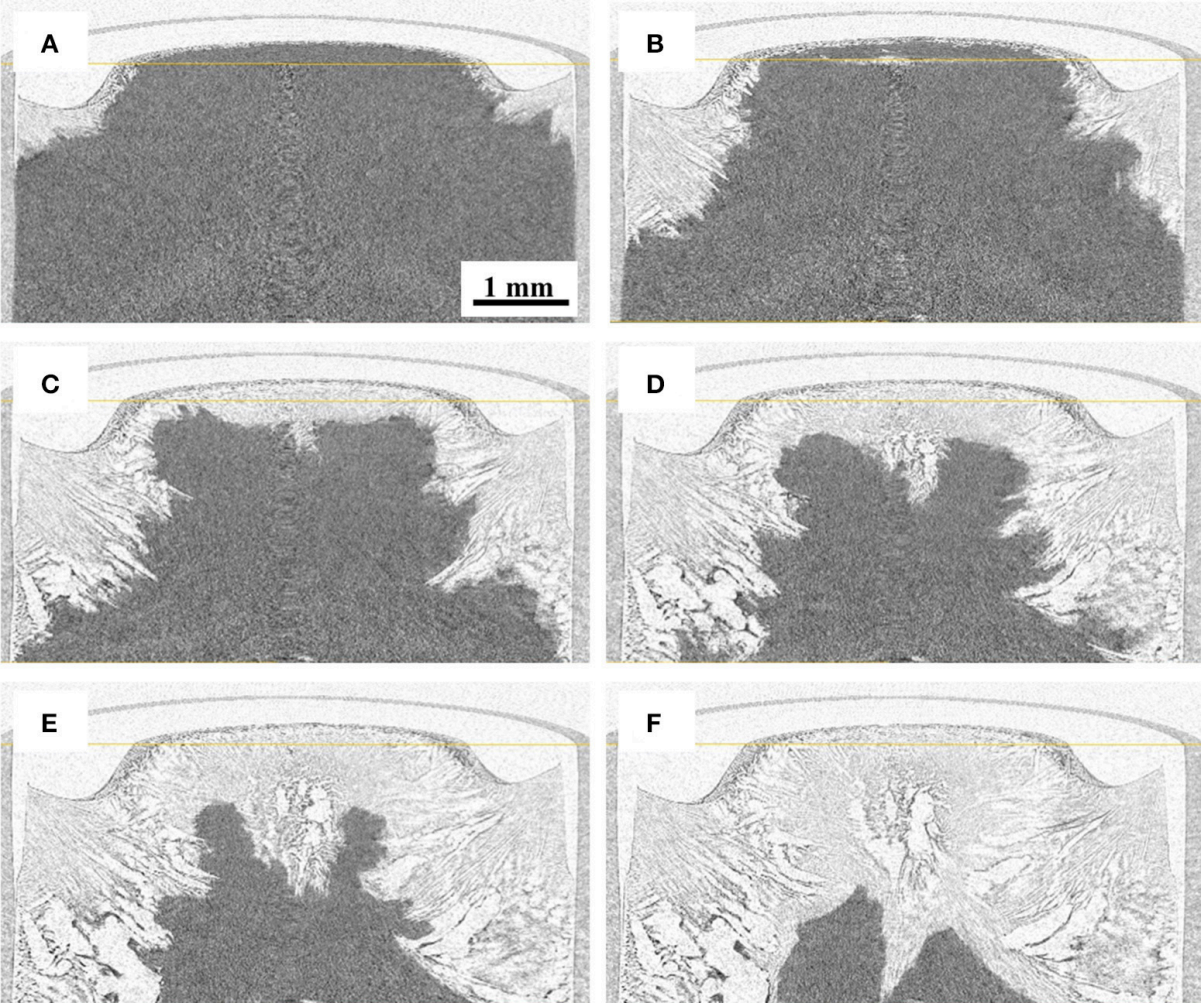
Three-dimensional (3D) X-ray CT images during freeze-drying of the non-annealed samples, dried for **(A)** 600, **(B)** 1,200, **(C)** 1,800, **(D)** 2,400, **(E)** 3,000, and **(F)** 3,900 s.

**Figure 5:**
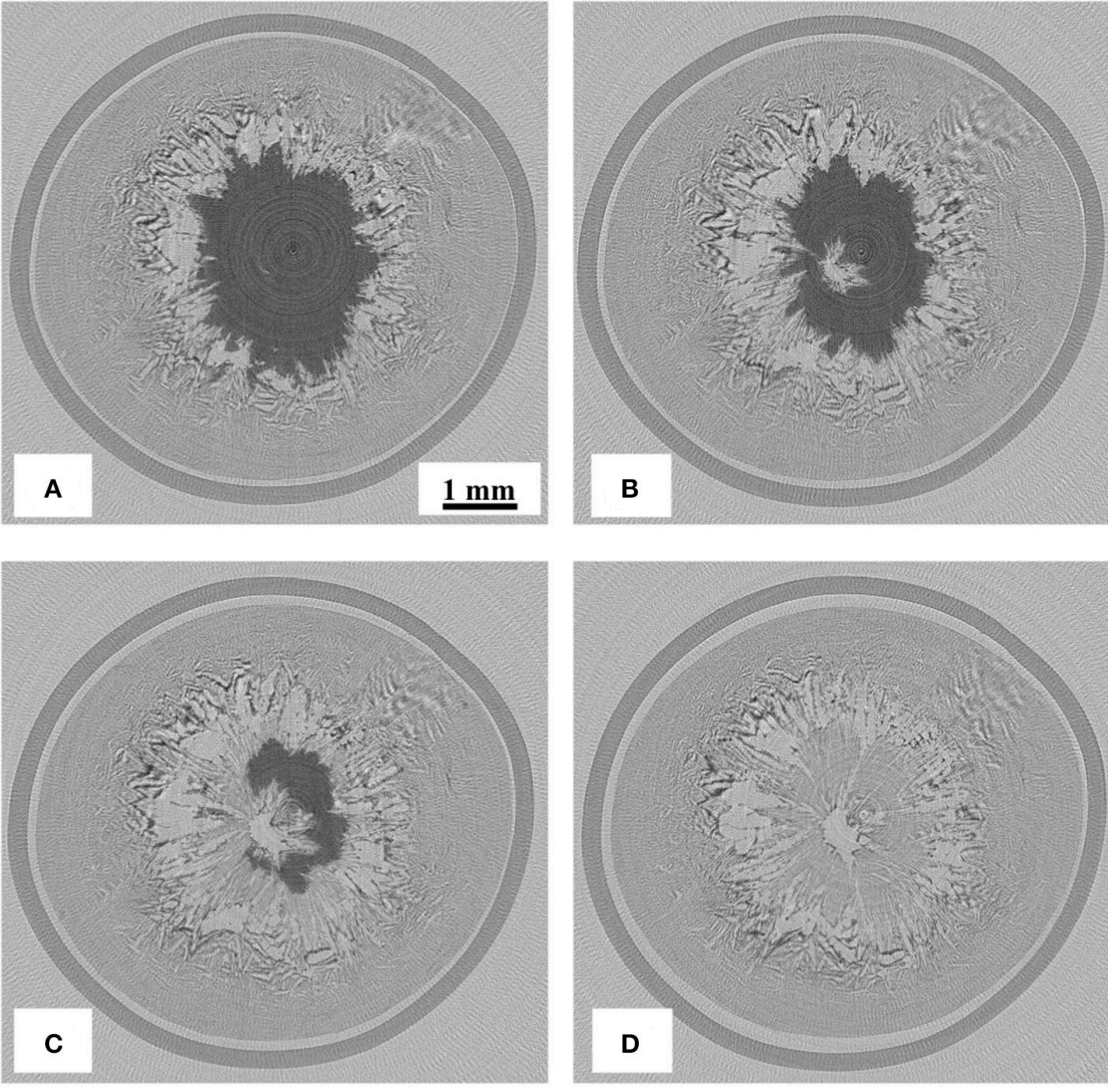
Two-dimensional (2D) X-ray CT images during freeze-drying of the non-annealed samples (horizontal cross-section at 900 μm from the top), dried for **(A)** 1,800, **(B)** 2,400, **(C)** 3,000, and **(D)** 3,900 s.

**Figure 6:**
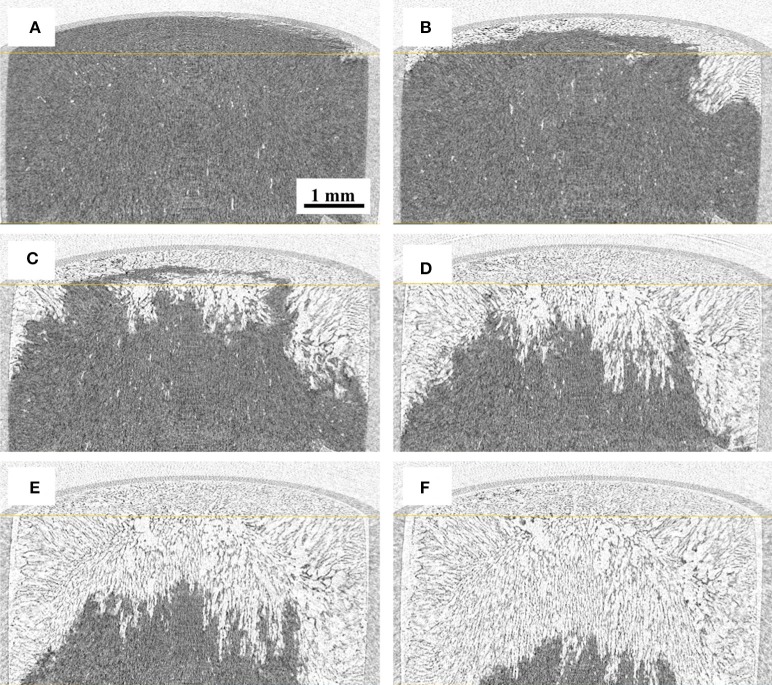
3D X-ray CT images during freeze-drying of the samples annealed for 12 h, dried for **(A)** 600, **(B)** 1200, **(C)** 1,800, **(D)** 2,400, **(E)** 3,000, and **(F)** 3,900 s.

**Figure 7:**
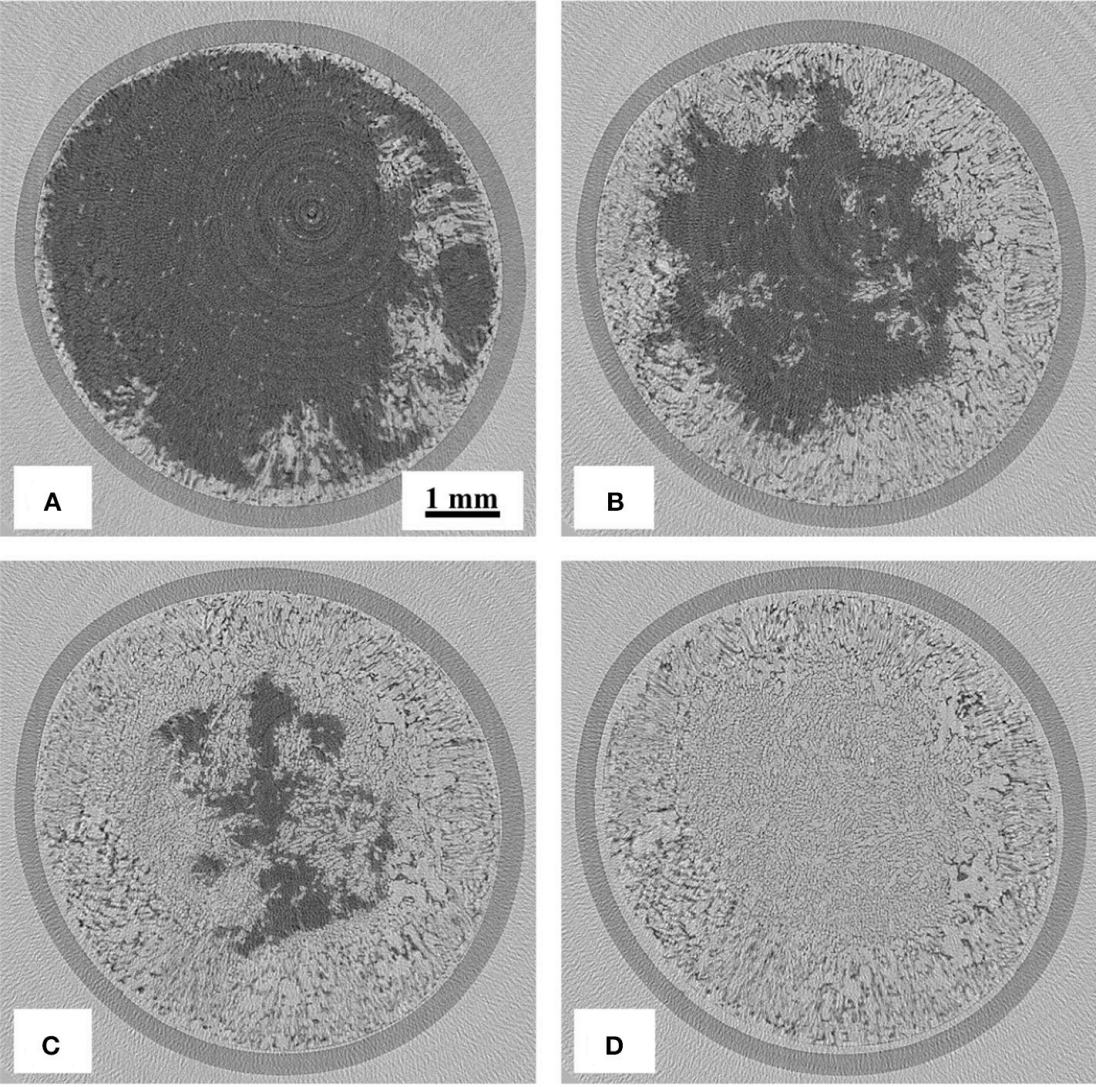
2D X-ray CT images during freeze-drying of the samples annealed for 12 h (horizontal cross-section at 1,500 μm from the top), dried for **(A)** 1,800, **(B)** 2,400, **(C)** 3,000, and **(D)** 3,900 s.

Reconstructed images of the samples during freeze-drying are shown in Figures 4–7, where the progress of the freeze-drying is clearly visualized. As in the case of the ice sublimation test (Figure 2), the sublimation interface did not perfectly one-dimensionally moved toward the bottom owing to the radiative heat from the housing material. Although the difference in the ice microstructure formations between the non-annealed and annealed samples was obvious, a significant difference in the drying rate was not observed in these images. Formation of extremely large pores was a notable feature in the sample prepared without annealing (Figures 4, 5). These pores differ from the majority of other smaller pores in terms of size and morphology. It was visibly confirmed from the images of the sample obtained with 12 h of annealing that the ice crystals were removed from the system while maintaining the original architectures attributed to the freeze-concentrated phase (Figures 6, 7). This observation agrees with the conventional explanation of a freeze-drying mechanism where a microstructure in a freeze-dried matrix is a replica of the frozen microstructure. On the other hand, this was not clearly observed in the images of the non-annealed sample (Figures 4, 5). Although extremely fine ice microstructures were formed in the frozen matrix, the original ice that could replicate the large pores was not confirmed. Considering that the glassy phase in the non-annealed solution was not perfectly freeze-concentrated, the apparent vapor pressure of this phase is higher than that of the perfectly freeze-concentrated phase. The apparent water vapor pressure (which is not an equilibrium thermodynamic pressure) of the glassy phase is lower around the glass transition temperature as the properties of the glassy phase are kinetically controlled (Slade et al., 1991). Therefore, in the drying of the non-annealed solution, the water removal from this phase is rapid (as the *T*_g_ is lower than the *T*'_g_), which could easily lose its original microstructure. These observations suggest that the microstructure of the freeze-dried matrix is not a perfect replica of the ice microstructures in the frozen solution; some parts of microstructure formations are due to the removal of water. The frequency of this microstructure formations could be affected by the properties of the glassy phase and rapidity of the drying.

### Image analysis of freeze-dried microstructure

**Figure 8:**
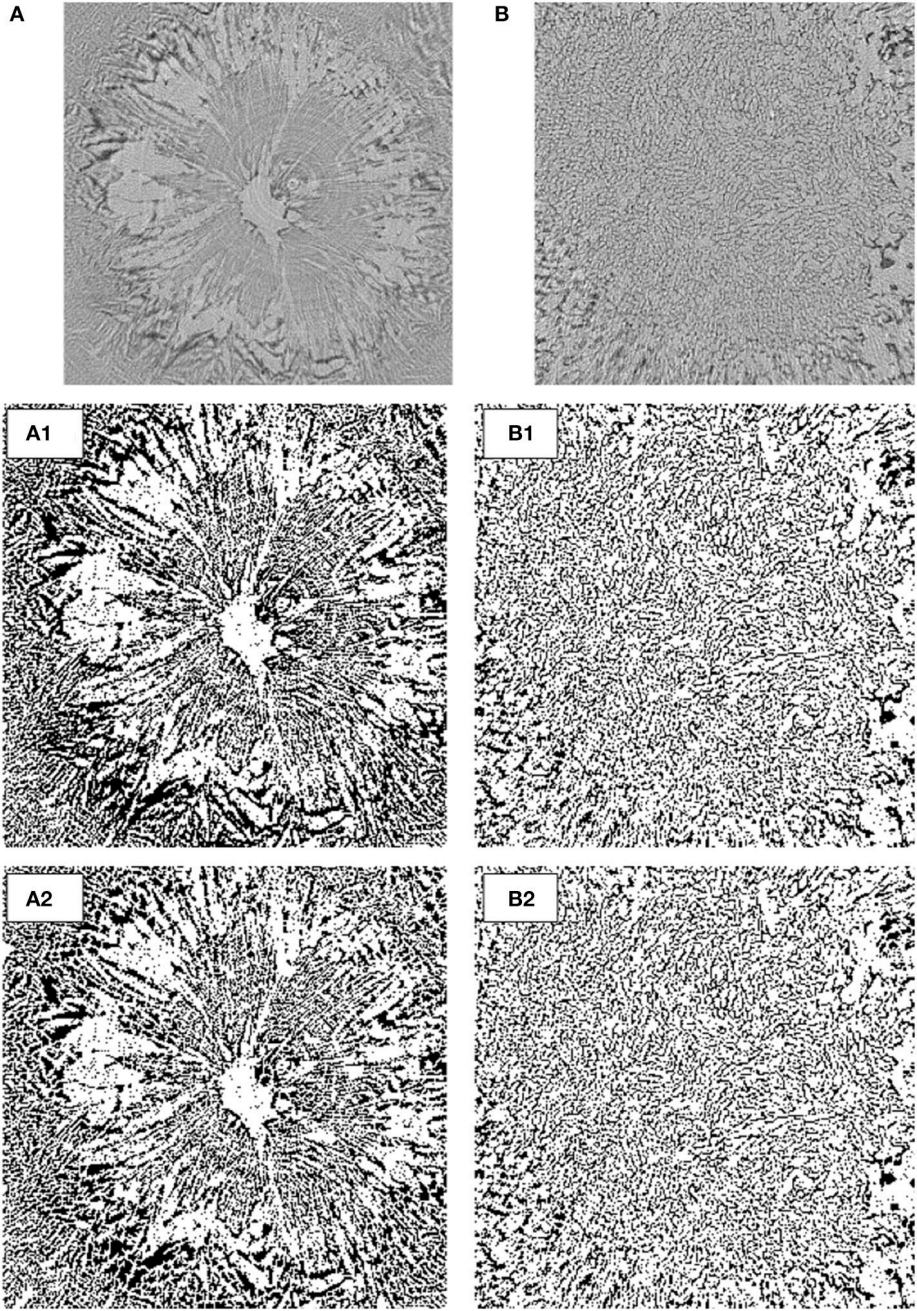
Binary images converted from cross-sectional CT images: **(A)** extracted from Figure 5D, **(B)** extracted from Figure 7D, (1) binary image, and (2) segmented by the watershed algorithm.

In order to evaluate the differences in freeze-dried microstructures, image analyses were performed (Figure 8). Cross-sectional images were selected from the dried zone in the images at 3,900 s of freeze-drying (Figures 5D, 7D), and converted into binary images for further image processing using ImageJ1.40 and FracLac version 2015 plug-in software.

First, the fractal dimension and lacunarity values of the porous microstructures were calculated from the images. Fractal dimension is a measure of structural roughness, whereas lacunarity is a measure of inhomogeneity or texture. In other words, lacunarity is the ratio of void space filled in a fractal structure. The fractal dimension values for the non-annealed and annealed samples were calculated to be 1.82 and 1.79, respectively. This implies that their fractal microstructures were almost equivalent. On the other hand, the lacunarity values for the non-annealed and annealed samples were 0.16 and 0.13, respectively. The difference in the lacunarity values suggests a difference of the morphological inhomogeneities in the fractal microstructures. The structural difference between the non-annealed and annealed freeze-dried matrices was mainly due to the formation of the void space. Therefore, the difference in the lacunarity values implies that the voids formed in the non-annealed and annealed samples were not equivalent.

Second, the binary images were segmented by the watershed algorithm (Figure 8C), and the mean diameters of the segmented fractions were compared. When water is removed from a frozen microstructure and the replicas of the ice crystals combine with each other to produce new pore microstructures, the freeze-concentrated region is supposed to be piled up to the pore walls, which consequently thickens the pore walls. This could be measured by estimating the mean size of the segmented fractions. The mean diameter values of the segmented fractions for the non-annealed and annealed samples were estimated to be 13.5 and 8.6 μm, respectively. This clearly supports the pore microstructure formation by the above mechanism. The ice microstructure of the rapidly frozen and non-annealed solution was extremely fine, and the glassy phase was not perfectly freeze-concentrated. Upon freeze-drying, pore microstructures formed as a consequence of the water removal. The pores were replicas of the original ice microstructures; some pore microstructures newly formed at the moment of water removal. When annealing was applied, the pore formation by the latter mechanism was minor. Annealing in the freeze-drying process has been recognized as a useful technique to reduce the primary-drying time and alter the crystallinity and/or aggregation degree of the components (Ablett et al., 1992; Sahagian and Goff, 1994; Milton et al., 1997; Kasper et al., 2013; Goshima et al., 2016). The results of this study suggested that these advantages of annealing could be realized by avoiding the deformation of the ice microstructures observed in the freeze-drying of the non-annealed sample.

## Conclusions

*In-situ* CT with X-rays from synchrotron radiation was used to observe microstructure formation during freeze-drying. A freeze-drying stage was designed and equipped at the X-ray CT stage. The frozen and dried microstructures of dextrin solutions were successfully observed. The images of the frozen samples confirmed that the ice crystal sizes increased with the annealing time. The boundary between the ice and freeze-concentrated phases became obvious after the annealing. This could be attributed to Ostwald ripening and glassy phase relaxation. In this study, annealing was applied to solutions that were frozen much rapid than a conventional freezing conditions (*ca* slower than 1.0°C/min). So in the case of conventional freezing, phenomena that could occur during annealing (i.e., glassy phase relaxation) simultaneously occur during freezing. A combination of rapid freezing and long annealing would thus lead similar result to a combination of slow freezing and short annealing. Observations of microstructure formation during freeze-drying revealed that the pore microstructures formed as a consequence of dehydration. The pore microstructures were replicas of the original ice microstructures; some pore microstructures newly formed by the removal of water. The latter mechanism was obvious in the non-annealed sample, suggesting that this mechanism is related to the relaxation level of the glassy phases. The glassy phase in the non-annealed solution was not perfectly freeze-concentrated. Therefore, water was rapidly removed from this phase, which lost its original microstructure. The occurrence of pore formation by this mechanism could be evaluated by estimating the mean pore thickness values by analyzing the CT images. When water was removed from a frozen microstructure and the replicas of the ices combined with each other to produce new pores, the freeze-concentrated region piled up to the pore walls, thus thickening the pore walls. These observations suggested that the advantages of annealing are not only to reduce the drying time owing to the modification of ice crystal morphologies but also to avoid quality loss related to the structural deformation of the glassy matters.

## Author contributions

KN made major contribution to this study on planning, experiment, analysis, and writing manuscript. ST and SS contributed to carry out experiments and data processing. GD and SK contributed to carry out experiments and image analysis.

### Conflict of interest statement

The authors declare that the research was conducted in the absence of any commercial or financial relationships that could be construed as a potential conflict of interest. The reviewer SG and handling Editor declared their shared affiliation.
